# 591 Transition to an Advanced Practice Provider led burn service: process and lessons learned

**DOI:** 10.1093/jbcr/irac012.219

**Published:** 2022-03-23

**Authors:** Maryke Bard, Lauren Lewis, Christopher Ward, Anna Frabotta, Richard B Lou, John Crow, Anjay Khandelwal

**Affiliations:** Akron Children's Hospital, Akron, Ohio; Paul and Carol David Foundation Burn Institute; Akron Children's Hospital, Akron, Ohio; Akron Children's Hospital Paul and Carol David Foundation Burn Institute, Akron, Ohio; Akron Children's Hospital, Twinsburg, Ohio; Akron Children's Hospital, Akron, Ohio; Akron Children's Hospital, Akron, Ohio; Akron Children's Hospital, Akron, Ohio

## Abstract

**Introduction:**

Burn is not a required rotation for general surgery residency training. Additionally, mandates on resident work hours have created a deficiency in resident support for many burn programs across the country. Subsequently, the need to transition to an Advanced Practice Provider (APP) service was identified. At our institution, a medium volume (~250 admissions/year) burn center, we created an APP service and evaluated the evolution and lessons learned during the transition.

**Methods:**

Timeline of recruitment of APPs was reviewed along with the recruitment process, onboarding process and orientation process. Exit interviews were conducted with all APPs who resigned to delineate strengths and weakness of the APP program. Functionality of the institution’s APP recruiter, manager and Lead Burn APP (LAPP) were reviewed as well.

**Results:**

Between 2014 and 2021 the daytime burn APP service expanded from two to six APPs and the Night House Officer (NHO) APP service was created and expanded to four APPs. Based on thorough review of the APP program, key changes were made including improvements in team communication, daily nurse-driven rounds, the 90-day focused professional performance evaluation (FPPE), enhanced and structured education, and critical care exposure.

The need for a LAPP was identified and designated in 2019. The LAPP is responsible for making an equitable schedule, execution of new APP orientation, organizing the 90 day FPPE and ongoing professional performance evaluation (OPPE), monthly APP peer review and APP meetings, coordinating OR coverage, and functioning as an APP advocate at division leadership. The LAPP enacted changes to the existing hiring/onboarding process as described below.

All APPs are now recruited through an APP recruiter who discusses the hospital contract and salary as well as organizes the logistics of the interview, formalizes the offer, and aids with the transition to credentialing. All new APPs are given a hospital onboarding checklist, that includes hospital orientation, APP boot camp, simulation and electronic health record training classes. In addition they are given a clinical orientation checklist, which includes BLS/ABLS/ACLS/ATLS certification, exposure to various phases of patient care, tracking of quantitative patient care metrics, participation in monthly burn APP peer review and burn triage simulation. There are subtle differences with the NHO APP clinical checklist. After 90 days of employment a new employee FPPE is held with the LAPP and burn director. This allows for direction of areas to focus on, comprehensive feedback, and discussion about increasing autonomy.

**Conclusions:**

Through a focused process, APPs can play an integral role in the daily functioning of a burn center. We believe that a similar effective and financially sound model can be created for similar sized units.

